# Protective Immunity to Dengue Virus Induced by DNA Vaccines Encoding Nonstructural Proteins in a Lethal Challenge Immunocompetent Mouse Model

**DOI:** 10.3389/fmedt.2020.558984

**Published:** 2020-10-30

**Authors:** Rúbens Prince dos Santos Alves, Robert Andreata-Santos, Carla Longo de Freitas, Lennon Ramos Pereira, Denicar Lina Nascimento Fabris-Maeda, Mônica Josiane Rodrigues-Jesus, Samuel Santos Pereira, Alexia Adrianne Venceslau Brito Carvalho, Natiely Silva Sales, Jean Pierre Schatzmann Peron, Jaime Henrique Amorim, Luís Carlos de Souza Ferreira

**Affiliations:** ^1^Laboratório de Desenvolvimento de Vacinas, Departamento de Microbiologia, Instituto de Ciências Biomédicas, Universidade de São Paulo, São Paulo, Brazil; ^2^Laboratório de Interações Neuroimunes, Departamento de Imunologia, Universidade de São Paulo, São Paulo, Brazil; ^3^Laboratório de Microbiologia, Centro das Ciências Biológicas e da Saúde, Universidade Federal Do Oeste da Bahia, Barreiras, Brazil

**Keywords:** mouse model, dengue, nonstructural proteins, DNA vaccines, IFN-γ

## Abstract

Dengue virus represents the main arbovirus affecting humans, but there are no effective drugs or available worldwide licensed vaccine formulations capable of conferring full protection against the infection. Experimental studies and results generated after the release of the licensed anti-DENV vaccine demonstrated that induction of high-titer neutralizing antibodies does not represent the sole protection correlate and that, indeed, T cell-based immune responses plays a relevant role in the establishment of an immune protective state. In this context, this study aimed to further demonstrate protective features of immune responses elicited in immunocompetent C57BL/6 mice immunized with three plasmids encoding DENV2 nonstructural proteins (NS1, NS3, and NS5), which were subsequently challenged with a DENV2 strain naturally capable of inducing lethal encephalitis in immunocompetent mouse strains. The animals were immunized intramuscularly with the DNA vaccine mix and complete protection was observed among vaccinated mice. Vaccine induced protection correlated with the cytokine profiles expressed by spleen cells and brain-infiltrating mononuclear cells. The results confirm the pivotal role of cellular immune responses targeting nonstructural DENV proteins and validate the experimental model based on a DENV2 strain capable of infecting and killing immunocompetent mice as a tool for the evaluation of protective immunity induced by anti-DENV vaccines.

## Introduction

Dengue fever is an acute disease caused by dengue virus (DENV), an arbovirus belonging to the *Flaviviridae* family transmitted by *Aedes* mosquitoes (1–4). Any of the four DENV serotypes can induce different degrees of illness, which are classified by the WHO as dengue fever, dengue with warning signs, and severe dengue (5). In fact, it is estimated that 3.9 billion people in 128 countries are at risk of infection (6). In addition, annually, 96 million DENV-infected people develop symptoms with sufficient severity to change their routine (7), and previous studies showed that ~500,000 individuals develop severe forms of disease, which may include hemorrhagic shock syndrome (8). The mortality rate in this group reaches 10% in hospitalized patients and 30% in nonhospitalized DENV-infected individuals (9). Regardless of the high epidemiological importance of dengue fever, there is no effective drug against the virus or a completely safe and widely available vaccine capable of preventing viral infection when used in endemic areas (10).

One of the main obstacles in understanding the disease and for the faster development of safer vaccines and/or anti-viral drugs is the lack of appropriate experimental models. Wild-type mice are usually resistant to infection with wild DENV strains since the virus is unable to block type I and type II interferon (IFN) receptor signaling in murine cells (11). Thus, most murine models for mimicking DENV infection are based on mouse strains with a defective immune system (12–15). These models have been useful to characterize the virus infection pathways and pathogenesis mechanisms since a clear infection phenotype is observed in these models, but they are unable to provide a comprehensive and accurate understanding of the natural immune responses induced by DENV infection. On the other hand, mouse-adapted virus strains, such as the DENV2 New Guinea C (NGC) strain, require the use of nonphysiological infection sites, such as the intracranial route, which raises several doubts about the immune mechanisms underlying the natural infection process (16–19).

Several vaccine formulations for dengue are currently under study, including vaccines with attenuated viral particles or chimeric viral proteins and DNA vaccines (20–24). Nonetheless, a tetravalent vaccine formulation requires simultaneous balanced and long-lasting immune responses against all four DENV serotypes. Otherwise, a vaccine that induces a poor or an imbalanced response to structural proteins of any of these viruses may possess a risk of virus replication enhancement, the antibody (Ab)-dependent enhancement (ADE) that occurs upon the presence of sub-neutralized antibody responses (25–28). In fact, epidemiological studies and phase III clinical trial data on Dengvaxia®, the only presently available licensed DENV vaccine, support the emergence of ADE, particularly for infants not previously exposed to DENV (29–31). In contrast, several mouse model studies have demonstrated that both virus-specific and cross-reactive T cells can confer immune protection to DENV (32–35). Indeed, the approach based on structural proteins as the only vaccine antigen target is probably the main reason why this anti-DENV vaccine did not succeed in inducing long-lived effective and safe protective immunity (10, 36).

Various studies have addressed the role of broadly reacting T cell responses to different DENV serotypes (34, 37–40). These studies led to the identification of responsive epitopes within the virus proteome and correlations with reactive HLA groups (41, 42). Studies based on HLA transgenic mice and human samples have identified several immune epitopes capable of conferring protective immunity and disclosed immunodominance drifts to specific HLA alleles in the human population (39). Moreover, CD8^+^ T cell responses target predominantly nonstructural (NS) proteins following infection with DENV in both mice and humans (34, 39, 43). Notably, CD8^+^ T cell responses target both structural and NS proteins following primary and homotypic secondary infection in both mice and humans (43). Nonetheless, CD8^+^ T cell responses target mainly conserved NS proteins following heterotypic secondary infection and vaccination with live attenuated DENV (34, 44) and during convalescence to natural infections (45–47).

DENV NS proteins are promising antigen candidates for high T cell-based immunogenicity, as demonstrated both by infection of nonhuman primates and by immunization with monovalent formulations based on NS proteins in a murine model (17, 19, 48, 49). Moreover, volunteers who received a mono- or tetravalent live-attenuated DENV vaccine developed broad responses to structural and nonstructural proteins after monovalent vaccination (34), which was also observed in patients experiencing secondary DENV infection (47). Additionally, T cell responses following tetravalent vaccination were dramatically focused on the highly conserved NS3 and NS5 proteins (34). Interestingly, several groups have previously shown separately the immunogenic and protective roles of NS1, NS3, and NS5 as antigen targets in vaccine candidates validated in murine models (20, 49, 50).

In the present study, we addressed the role of T cell responses against DENV NS proteins using a mix of DNA vaccines encoding DENV2 NS1, NS3, and NS5. In addition, the DNA vaccines were tested with a new infection model based on a nonadapted neurovirulent DENV2 strain (JHA1) capable of inducing encephalitis and death in immunocompetent mice. The present results confirm the role of DENV NS proteins as relevant antigen targets for T cell responses and demonstrate the usefulness of the proposed experimental lethal challenge immunocompetent mouse model for testing anti-DENV vaccines.

## Materials and Methods

### Animals and Ethics Statement

Five- to six-week-old male mice were maintained under SPF conditions at the Isogenic Mouse Facility of the Parasitology Department, University of São Paulo, Brazil. All mice were used according to the Brazilian College of Animal Experimentation (CONEP) guidelines, and the Institutional Animal Care and Use Committee (CEUA) of the University of São Paulo (protocol number 14/2016) approved the protocols.

### Viruses, Cell Lines, and Plaque Assay

Triple plaque-purified DENV2 strain JHA1 (18, 51) was propagated in C6/36 cells cultured in Leibovitz (L-15) medium (Gibco, USA) supplemented with 2% fetal bovine serum (FBS) (Gibco, USA) and quantified by plaque assay carried out with Vero cells cultured in DMEM (Embriolife, Brazil) supplemented with 10% FBS, as previously described (18). Their titers are given in plaque-forming units (PFU). VERO cells were cultivated in DMEM (Embriolife, Brazil) supplemented with 10% FBS. The genome of the virus used in this work has been deposited in GenBank under the accession no. JQ686088.2. To access viral load in the brain, mice were euthanized by CO2 inhalation and, then, the brains were harvested after extensively perfusion with PBS. The organ was, then, macerated with 2 mL of DMEM medium supplemented with 2% FBS and antibiotics. After centrifugation, 1 mL of each supernatant was serially diluted, added to VERO cells monolayer, and the titers were determined as described above. Titers were expressed as the log PFU/mL.

### Characterization of a Lethal Infection With JHA1 in C57BL/6 Mice

Five-week-old C57BL/6 (B6 WT) mice were divided into two groups (*n* = 5-8), which were administered intracranially (i.c.) with 1x10 (2) PFU diluted to a final volume of 40 μL with L-15 medium. A mock group was injected with L-15 medium alone. The animals were monitored daily for mortality, changes in body weight, pathological symptoms and serum biochemical markers. The body weight loss was calculated daily for each animal until the day of death or the conclusion of the monitoring period and is represented as the percentage of the final weight compared to the initial weight. On days 0, 1, 3, 5, and 7 after the infection, animals were bled via the submandibular vein for individual determination of serum levels of aspartate aminotransferase (AST), alanine aminotransferase (ALT), and lactate dehydrogenase (LDH) determined with an analytical kit as recommended by the supplier (Laborclin, Brazil).

### Immunohistochemistry

Paraffin-embedded PFA-fixed mouse brain sections were deparaffinized with xylene and rehydrated. One of two consecutive sections was stained with hematoxylin and eosin (H&E), and the other was subjected to immunofluorescence staining. Antigen retrieval was performed by boiling in 0.1 M citrate buffer pH 6 for 10 min. Tissue was permeabilized in 0.1% Triton X-100 in PBS for 20 min and blocked in 10% FBS for 30 min. Further staining included anti-flavivirus mouse monoclonal 4G2 and a secondary anti-mouse IgG conjugated with Alexa Fluor® 568 (1:100, Thermo Fisher). Cells were visualized under a fluorescence microscope (EVOS FI).

### DNA Vaccines and Transfections

The customized pNS1, pNS3, and pNS5 plasmids encoding *ns1, ns3* and *ns5* from the DENV2 JHA1 strain (GenBank JQ686088.2), respectively, were purchased from GenScript, USA. The NS1 and NS5 sequences were cloned in a pVAX-1 vector (Novagen, USA) using the restriction sites for *BamHI* and *XhoI* at the 3′ and 5′ extremities, respectively. The NS3 sequence was cloned in pVAX-1 using the restriction sites for *EcoRI* and *XhoI* at the 3′ and 5′ extremities, respectively. BHK cells were transiently transfected with the DNA vaccines pNS1, pNS3, and pNS5 or the control plasmid pVAX-1, as previously described (52). Briefly, 2 × 10^5^ cells/well were plated in 12-well plates (Nunc, Denmark) with Opti-MEM medium (Invitrogen) and transfected with 1.2 μg of each DNA plasmid using lipofectamine (Invitrogen) under conditions suggested by the manufacturer. Cell monolayers were then maintained for 48 h at 37°C with 5% CO_2_. On the following day, the cells were washed in 0.1 M phosphate buffer pH 7.4, fixed in 1% formaldehyde for 20 min, permeabilized with 0.6% saponin (for pNS1 and pNS3) or 0.1% Triton X-100 (for pNS5) for 10 min and blocked with 1% bovine serum albumin (BSA) (Sigma) and 0.2% saponin or 0.1% Triton X-100 for 15 min. Cells were then incubated for 1 h at 37°C with the anti-NS1 mab 4F6 or anti-NS5 mab GT361 (Genetex, USA) diluted 1:1500 or anti-NS3 hyperimmune mouse serum diluted 1:1000. The wells were then washed three times and incubated for 1 h at 37°C with fluorescein-conjugated goat anti-mouse IgG (Southern Biotechnology, USA) diluted 1:100. Cells were visualized under a fluorescence microscope (EVOS FI).

### Immunization and Challenge

Five- to six-week-old B6 WT mice were subjected to an immunization regimen of two doses of the vaccine formulation pNS (1, 3, and 5) composed of 50 μg of each plasmid (pNS1, pNS3, and pNS5) administered intramuscularly (i.m.) at a 15-day interval. A control group (*n* = 10) was injected with the backbone plasmid (pVAX) in endotoxin-free PBS (pH 7.4). Mice were bled from the facial vein before the first vaccine dose and two weeks after each round of immunization. Serum samples were collected after blood coagulation at 37°C for 30 min and at 4°C for 30 min, followed by centrifugation at 3,000 x g for 15 min at 4°C. Serum samples were stored at−20°C until testing. Serum samples were subjected to ELISA for detection of the IgG titers to recombinant versions of NS1, NS3 helicase domain, and NS5 proteins. These antigens were produced in *E. coli* and the assay was performed as previously described (17, 38). Briefly, 96-well Maxsorp™ ELISA plates (Costar) were coated with each recombinant protein (200 ng/well) in 100 μl coating buffer (PBS, pH 7.4) overnight at 4°C and then blocked for 1 h at room temperature (RT) with 3% skimmed-milk in PBS (Thermo Fisher Scientific). Mouse serum samples were serially diluted three-fold from 1:100 to 1:3,600 in block solution and added to the coated wells. As positive controls, we used anti-NS1 mab 4F6 or anti-NS5 mab GT361 (Genetex, USA) with the first dilution starting with 1 μg/ml, or diluted anti-NS3 hyperimmune mouse serum as indicated. After 1 h incubation at RT, the wells were washed with 0.05% Tween 20 in PBS and, then, incubated with HRP-conjugated goat anti-mouse IgG (Sigma-Aldrich, USA) (1:4000 in block solution) for 1.5 h at RT. After a final washing, plates were developed with sodium citrate buffer (pH 5.8) containing ortho-phenylenediamine dihydrochloride (Sigma-Aldrich, USA) and H_2_O_2_, and the reaction was stopped after 15 min with the addition of 50 μl of H_2_SO_4_ at 2 M. The optical density (O.D.) reading was performed at 492 nm with a plate reader (Labsystems Multiscan, ThermoScientific, USA). For the challenge assay, mice (*n* = 10) were i.c. challenged with the neurovirulent DENV2 JHA1 clinical isolate, as previously described (18). Mice were immunized with the isolated vaccine formulations in two doses [either 100 μg of each plasmid (**Figure 3**) or a mix of 50 μg of each NS-coding plasmid (total 150 μg of DNA)], with the same interval between doses, as that described above. The control group was injected with 100 μg of empty DNA vector. After two weeks following the last vaccine dose, mice were anesthetized with a mixture of ketamine-xylazine and then inoculated by the intracerebral route with 20 μL of a viral suspension corresponding to 1 x 10^2^ PFU of JHA1 DENV2. After the challenge, the mice were monitored daily for 15 days.

### Spleen Cell Isolation

Mice were euthanized with CO_2_ two weeks after the third immunization, and the spleens were removed aseptically, as previously described (17). Splenocytes were suspended and washed once with RPMI 1640 medium (Sigma) with 2% fetal bovine serum (GIBCO). Red blood cells were lysed with 3 ml of ACK solution (150 mM NH3Cl, 10 mM KHCO3, 0.1 mM EDTA) per spleen for 3 min at room temperature. After two additional washes with RPMI and 2% FBS, the spleen cells were resuspended in R10 medium (RPMI supplemented with 10% FBS). Cell viability was assessed using 0.1% trypan blue dye exclusion, and cells were counted using a Neubauer chamber.

### Brain-Infiltrating Mononuclear Cell Isolation

Separation of mononuclear cells was performed as previously described (53). All mice were euthanized in CO_2_ chambers and perfused with 10 mL of cold PBS. Brains were excised, macerated and maintained in 4 mL of HBSS medium with calcium and magnesium and supplemented with 2.5 mg/mL collagenase D (Roche®) at 37°C for 45 min. After 45 min, tissue suspensions were washed with 15 mL of HBSS without calcium and magnesium and supplemented with EDTA (0.5 mM) and centrifuged at 450 × g for 5 min at 4°C. The cells were then resuspended in Percoll® (Sigma) (25%) and gently laid over Percoll® (70%) in 15 mL tubes. The tubes were centrifuged at 900 × g for 20 min at 22°C with the centrifuge brakes turned off. After centrifugation, the interface containing mononuclear cells was collected, washed with HBSS without calcium and magnesium and centrifuged at 450 × g for 5 min. Cellular suspensions were then suspended in complete RPMI medium, counted and used as desired.

### *Ex vivo* IFN-γ ELISPOT Assays

As previously described (17), total spleen cells (2 x 10^5^ cells/well) were cultured in 96-well plates (Millipore, USA) previously coated with 10 μg/ml anti-IFN-γ capture antibody (BD Biosciences, USA). Cells were then incubated and stimulated or not with 100 ng of each peptide/well for 48 h. The sequences of the peptides derived from the DENV2 NS protein restricted to either H-2D^b^ or I-A^b^ (respectively) are NS1 (KMLSTELH; NQTFLIDGPETAECP), NS3 (SAIAQTEKSI; GKTKRYLPAIVREAI), and NS5 (RMLINRFTM; VKVLNPYMPSVIEKM). Cells were then discarded, and the plates were washed with PBS containing Tween 0.05% (PBST), followed by incubation with a biotinylated anti-mouse IFN-γ mab (BD Biosciences, USA). After 16 h, the plates were again washed and then incubated with 200 ng/well peroxidase-labeled streptavidin (BD Biosciences, USA) for 2 h at 25°C. After washing, spots were developed by incubation with a diaminobenzidine solution for 30 min. The spots were counted with the aid of a magnifying glass. The number of IFN-γ-producing cells was calculated after subtracting the number of spots in the respective unstimulated sample.

### Quantification of Cytokines by CBA

Two weeks after the last vaccine dose, the animals were euthanized and perfused exhaustively with PBS. Spleens and brains were removed and processed for collection of immune cells. For the isolation of mononuclear cells from the brains of the mice, a gradient was made in Percoll® according to a previously described protocol (54). Total spleen cells or mononuclear cells extracted from the brain (2 × 10^5^ cells/well) were cultured in 96-well plates (Nunc, Denmark). The cells were plated in duplicate and one of the wells was not used stimulus. For stimulation, either the pool of 6 peptides or the peptides derived from the protein use in the immunization were added as described above, always at a concentration of 100 ng per well of each peptide. The dosages of cytokines in the supernatant of the stimulated cultures were made using the Cytometric beads array (CBA) method using the Th1/Th2/Th17 kits (BD Biosciences, CA, USA), according to the manufacturer's instructions. The samples were read by flow cytometry using the LSR Fortessa device (BD Biosciences, CA, USA). The results were generated from BD CBS Analysis Software and the graphs and statistical analysis were generated with the GraphPad Prism Software.

### Data Analysis

All statistical significance was calculated using Prism 6 software (GraphPad Software). The statistical significance values (*p-value*) were determined using two-way ANOVA followed by a Bonferroni post-test. To compare means, the nonparametric Mann-Whitney test was used. Survival curves were compared using the log-rank test. All differences with *p* < 0.05 were considered statistically significant.

## Results

### JHA1 Infection Is Lethal to B6 WT Mice

The lack of an adequate animal model for dengue virus (DENV) infection is often referred to as a main obstacle to a better understanding of DENV pathogenesis and, consequently, delays in the development of efficient and safe vaccines and antiviral drugs (12, 13, 55). In this sense, we developed an immunocompetent mouse model using the non-adapted DENV2 JHA1 strain originally isolated from a symptomatic patient in Brazil for testing the protective immunity induced by anti-DENV vaccines (51). As previously described, this clinical DENV2 isolate was naturally capable of replicating, inflicting neuropathogenic signs and hematological disturbances, and killing i.c. infected BALB/C mice (18).

**Figure 1:**
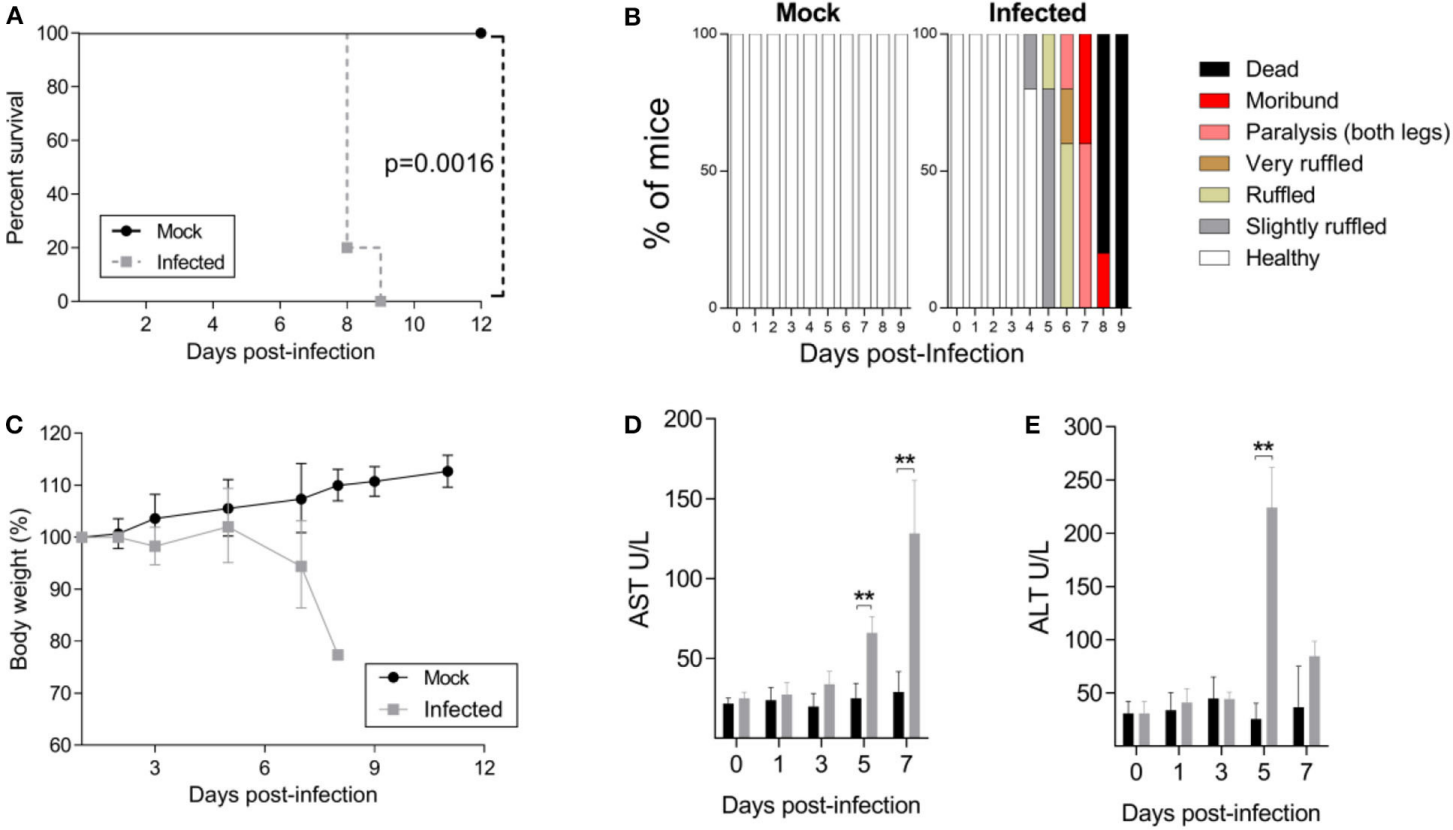
Characterization of the JHA1 i.c. lethal DENV challenge model. Four- to 5-week-old B6 WT mice were inoculated i.c. with 1 x 10^2^ PFU of JHA1 or vehicle (mock). The survival **(A)**, clinical scores **(B)**, and body weight **(C)** were examined. Mice having >20% weight loss and/or exhibiting paralysis of two hind limbs were euthanized according to the animal protocol. Levels of liver aminotransferases [AST **(D)** and ALT **(E)**] were measured in sera after challenge. In **(A,C)**, graphics were represented as pooled data from 2 different experiments with *n* = 8 mice per group. In **(B–D)**, data from a representative experiment are shown (*n* = 5). Data were analyzed by two-way ANOVA followed by Bonferroni's multiple comparisons test or log-rank test, where **p < 0.01.

In the present study, we determined whether the JHA1 strain would infect and kill adult B6 WT mice following i.c. inoculation. Five-week-old mice were injected with 1 × 10^2^ PFU of the JHA1 strain, which resulted in 100% infection-associated death 9 days after the challenge, while under the same conditions, no death was recorded among mock-treated mice (Figure 1A). Clinical signs associated with the infection started at day 4 post infection, with slightly ruffled fur followed by signs that escalated to a hunchback posture and tail and leg paralysis, reaching a higher degree of morbidity when the animals were euthanized (Figure 1B). Infected mice started to lose body weight 3 days after infection, and the weight loss progressed until day 7. Most of the infected mice lost ~20% of their initial body weight and showed a high degree of morbidity before death (Figure 1C). In addition, increased levels of AST and ALT detected 5–7 days after infection suggest liver damage in the infected animals (Figures 1D,E). Thus, these results demonstrated that the DENV2 JHA1 strain is lethal to adult B6 WT mice following i.c. inoculation.

### JHA1 Is Neurovirulent to C57BL/6 Mice

Neurovirulence can be defined as the ability of a virus to induce neurologic disease, and it is not necessarily dependent on neurotropism or neuroinvasion (56). Aiming to characterize the neuropathology of JHA1-infected animals, five-week-old mice were infected with JHA1 (1 x 10^2^ PFU) through the i.c. route, and seven days after the infection, histopathological analyses of the mouse brains were performed.

**Figure 2:**
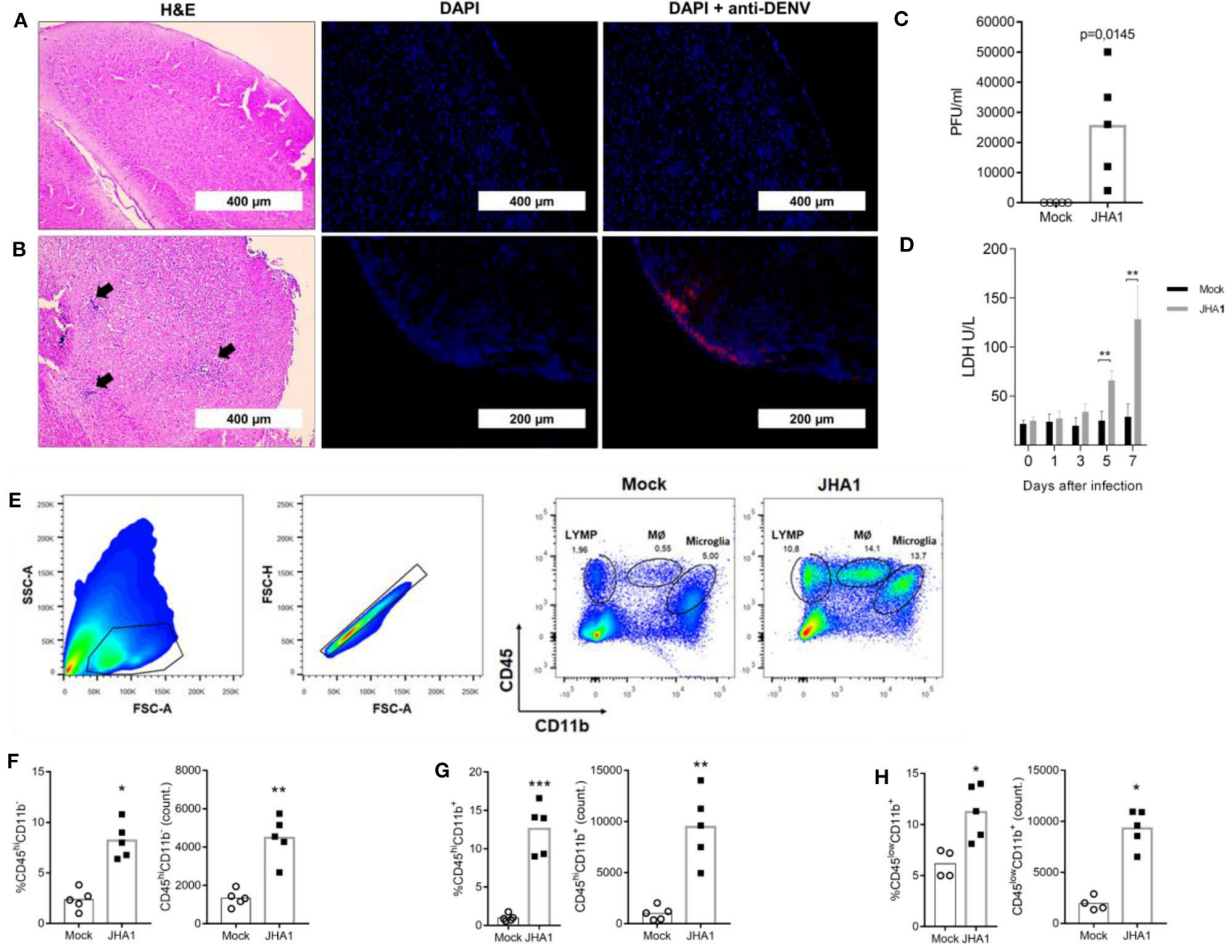
Immunohistopathological analyses of JHA1-infected mouse brains. Five- to 6-week-old B6 WT mice (*n* = 5), were inoculated i.c. with 1 x 10^2^ PFU of JHA1 or vehicle (mock). On day 7 after infection, brain sections of mock-infected **(A)** and JHA1-infected **(B)** mice were stained with H&E (**A,B**, left panels) or DAPI (blue) and anti-DEN antibodies (red) (**A,B**, middle and right panels). Black arrows indicate infiltration of cells with lymphocyte morphology. Viral load in the brain was assessed at day 7 post infection **(C)**. Levels of the general tissue damage marker lactate dehydrogenase (LDH) in the sera of mice were assessed prior to infection and at the 1st, 3rd, 5th, and 7th days post infection **(D)**. The gating strategy for the mononuclear infiltration analysis is exemplified in **(E)**. Graphs showing percentages and absolute numbers of **(F)** lymphocytes (CD45^high^CD11b^−^), **(G)** macrophages (CD45^high^CD11b^+^), and **(H)** microglia (CD45^low^CD11b^+^) are shown. Data were analyzed by two-way ANOVA followed by Bonferroni's multiple comparisons test or a nonparametric Mann-Whitney test, where *p < 0.05, **p < 0.01.

H&E-stained brain sections obtained from mice inoculated with the vehicle (control group) or virus (infected) were examined for the presence of infiltrating and infected cells (Figures 2A,B, respectively). The degree of infiltration appeared to be augmented in DENV-infected mice compared with that in control mice at day 7 post infection. Morphologically, the infiltrates showed mixed inflammatory cell populations, with numerous neutrophils in the tissue samples collected from infected mice. In addition, brain sections stained with fluorophore-conjugated flavivirus-specific 4G2 mab showed the presence of infected cells in DENV-infected brains (Figures 2A,B). Quantification of viable viral particles produced in the brain showed a significant increase in virus titers in this organ, an ~250-fold increase regarding the amount of initially inoculated viruses (Figure 2C). Under the testing conditions, the numbers of viable viruses in blood and other tissues were under the detection limit of the assay and similar to previous results described with Balb/c mice (18, 38) (Data not shown). Since DENV replication in organs is associated with tissue damage and the release of intracellular molecules, such as LDH, we quantified this biomarker in mouse sera following infection. As shown in Figure 2D, LDH levels increased over time, reaching significant increases in infected mice at days 5 and 7 after viral inoculation.

Aiming to more precisely define the infiltrating cells in brain tissues, we performed flow cytometry analyses with mononuclear cells isolated from infected brains. Using the gating strategy shown in Figure 2E, we assessed the frequency and absolute numbers of lymphocytes (CD45^high^CD11b^−^; Figure 2F), macrophages (CD45^high^CD11b^+^; Figure 2G), and microglia (CD45^low^CD11b^+^; Figure 2H). Enumeration of the inflammatory cells from individual brains confirmed the presence of different cell populations in DENV-infected mice with regard to those in mock-infected mice. These results indicate that JHA1 replicates in the brain tissue of B6 WT mice and induces inflammation in the central neural system (CNS), characterized by brain infiltration of inflammatory cells and tissue damage associated with viral replication.

### The DNA Vaccines pNS1, pNS3, and pNS5 Induce Immune Responses in WT B6 Mice

**Figure 3:**
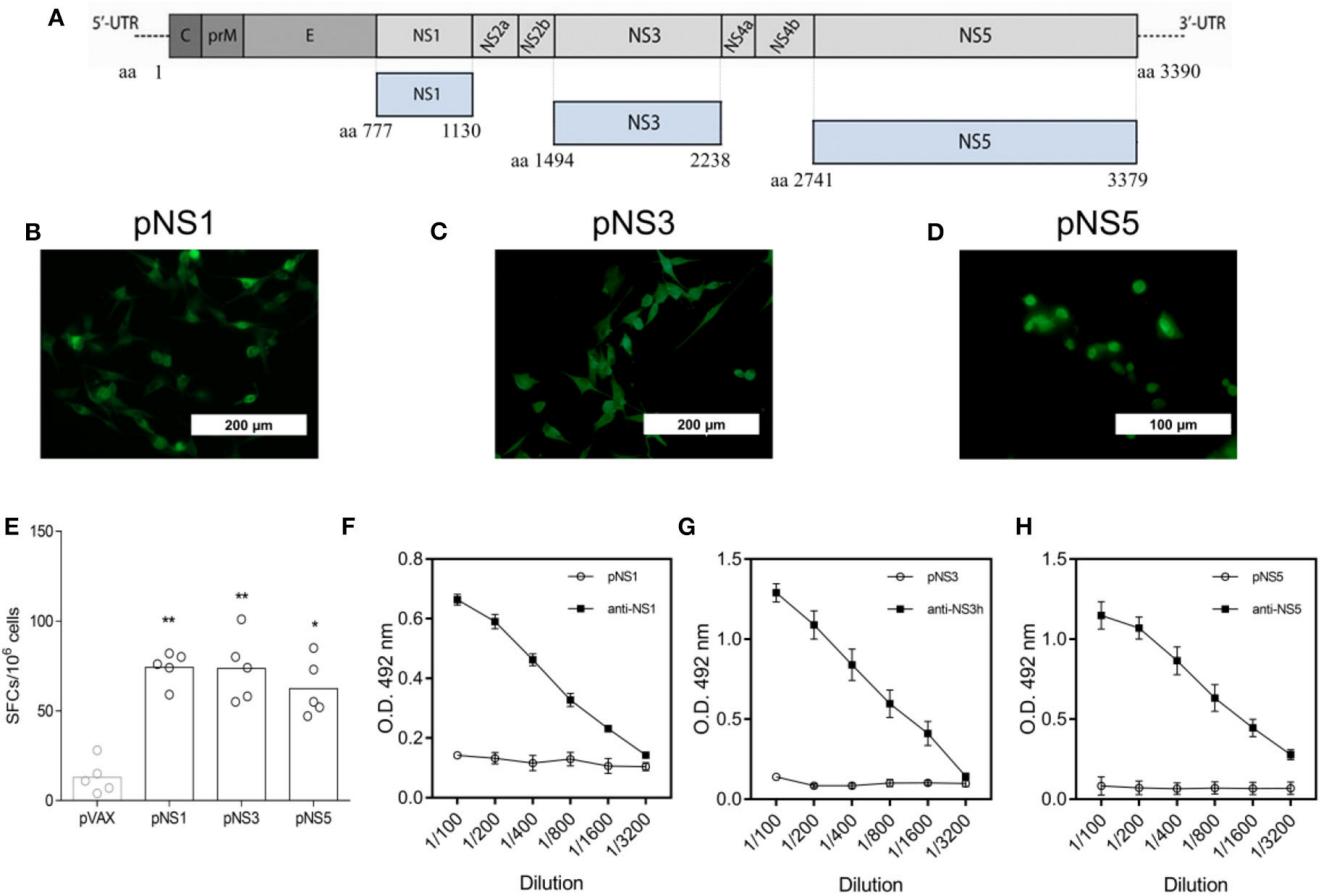
Schematic representation and immunogenicity of tested DNA vaccines encoding DENV2 NS1, NS3, or NS5. Gene sequences encoding the nonstructural proteins 1, 3, and 5 from DENV 2 (strain JHA1) were used to develop the DNA vaccines pNS1, pNS3, and pNS5, respectively **(A)**. BHK cells were transfected with each plasmid encoding NS1, NS3, or NS5, and protein expression was detected by anti-NS1 4F6 mab **(B)**, anti-NS3 polyclonal antibody **(C)**, or anti-NS5 GT361 mab **(D)**, followed by incubation with anti-mouse fluorescein-conjugated goat IgG. Splenocytes from mice (*n* = 5) immunized with two doses of each DNA vaccine were obtained two weeks after the last dose and stimulated with a pool of peptides from NS1, NS3, or NS5, and the result was assessed by IFN-γ ELISpot analysis **(E)**. Two weeks after the last dose of each vaccine, mice were bled, and sera were tested for the presence of DENV2 NS1, NS3h, or NS5-reactive IgG by ELISA (**F–H** respectively), using the recombination versions of DENV 2 full-length NS1 and NS5, as well the domain helicase of NS3. Statistical analyses were performed by a nonparametric Mann-Whitney test, where *P < 0.05 and **P < 0.01.

With the experimental immunocompetent mouse model in hand, we set conditions to evaluate the protective role of anti-DENV vaccines based on NS proteins. For that purpose, we tested a mix of three different DNA vaccines encoding NS1, NS3, and NS5 to induce specific T cell responses. The DNA vaccines encoding pNS1, pNS3, or pNS5 were generated based on the gene sequences of the DENV2 JHA1 strain (Figure 3A). To demonstrate the expression of the encoded proteins, BHK cells were efficiently transfected with each plasmid, separated, and submitted to immunofluorescence assays that allowed the detection of recombinant NS1 (rNS1) and rNS3 proteins in the cell cytoplasm, while detection of rNS5 was restricted to the cell nucleus, as previously reported (Figures 3B–D) (57). Detection of these proteins in transfected cells using specific monoclonal or polyclonal antibodies confirmed the expression and antigenicity of the target proteins.

To assess the immunogenicity of the DNA vaccines, mice were immunized separately with 2 doses of 100 μg (i.m.) of each plasmid. Vaccinated animals were euthanized 2 weeks after the second dose for splenocyte isolation, *in vitro* cultivation, and stimulation with NS (1,3,5)-derived synthetic peptides. All immunized mice, independent of the plasmid received, showed a higher number of IFN-γ-producing spleen cells than mock-treated control mice (Figure 3E). In contrast, vaccination with individual plasmids did not induced the production of antigen-specific IgG (Figures 3F–H). Hence, immunization with pNS1, pNS3, and pNS5 induced immune responses based mainly on T cells rather than antibody-mediated immune responses.

### Immunization With the DNA Vaccine Mix Induced Full Protection Against JHA1 Lethal Challenge in B6 WT Mice

**Figure 4:**
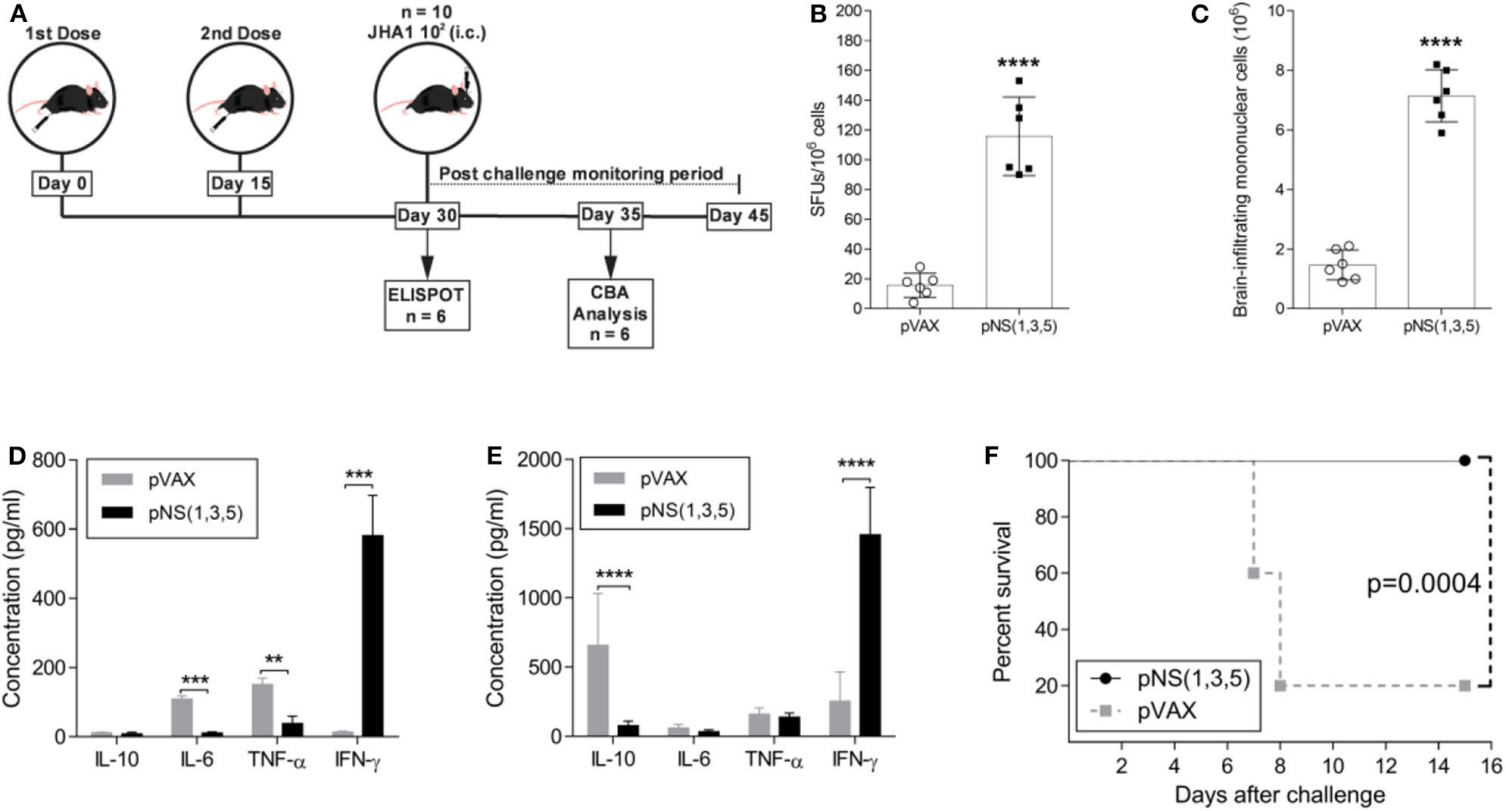
Characterization of vaccine-induced immune protective responses. Five- to six-week-old B6 WT mice were immunized with 100 μg of pVAX or 50 μg of each vaccine plasmid in 2 doses, with a fifteen-day interval between doses. The immunization regimen and the investigative approach are schematically represented in **(A)**. At day 30, mice were either submitted to ELISPOT analysis for IFN-γ production (*n* = 6) **(B)** or challenged with 1 × 10^2^ PFU of JHA1 (*n* = 10). Five days after challenge, the numbers of brain-infiltrating mononuclear cells were assessed **(C)**, and the production of IL-10, IL-6, TNF, and IFN-γ was quantified in the supernatant of brain-infiltrating mononuclear cells **(D)** or splenocytes **(E)** after stimulation with a pool of DENV2-derived peptides. The percent survival after challenge was monitored for 15 days **(F)**. The data were analyzed by a nonparametric Mann-Whitney test or by two-way ANOVA followed by Bonferroni's multiple comparisons test, where **p < 0.01, ***p < 0.001, and ****p < 0.0001. Survival curves were compared using the log-rank test.

Since immunization with each plasmid generated IFN-γ-producing T cells, we assessed the immunogenicity and protective immunity with the simultaneous administration of the three DNA vaccines. Therefore, 5- to 6-week-old mice received either two doses of a vaccine mix containing 50 μg of each vaccine plasmid [pNS (1, 3, 5)] or 100 μg of the backbone vector (pVAX), with a two-week interval between doses (Figure 4A). To assess the immunogenicity of the vaccine formulation, we analyzed IFN-γ production using spleen cells from immunized animals with peptides derived from the antigens (NS1, NS3 and NS5) for stimulation in ELISPOT two weeks after the last vaccine dose (Figure 4B). The number of IFN-γ-producing spleen cells after DENV-derived peptide stimulation was significantly higher in the pNS (1,3,5)-vaccinated group than in the control group (~9-fold increase; *p* < 0.0001). Since i.c. infection with JHA1 induced infiltration of inflammatory cells into the brain, we determined whether immunization with pNS (1,3,5) would impact the number of brain-infiltrating mononuclear cells. At day 5 post challenge, the vaccinated group had ~5-fold more brain-infiltrating cells (*p* < 0.0001) than the control group (Figure 4C). Furthermore, these cells showed an enhanced cytokine production profile after *ex vivo* DENV-specific stimulation.

Following stimulation of brain-infiltrating mononuclear cells with DENV NS-derived peptides, brain-infiltrating cells from vaccinated mice produced mainly IFN-γ (~42-fold more than control group; *p* < 0.001) (Figure 4D), whereas those from vector-immunized mice produced IL-6 and TNF. Interestingly, under the same experimental conditions, spleen cells from vector-immunized mice produced mainly the anti-inflammatory cytokine IL-10 (~9-fold more than vaccinated group; *p* < 0.0001), while animals immunized with the DNA vaccines encoding the NS proteins showed robust production of IFN-γ (~6-fold more than control group; *p* < 0.0001) (Figure 4E). To determine protection against DENV2, immunized mice were challenged with JHA1; 100% of mice immunized with pNS (1,3,5) were protected, and only 20% in the pVAX-immunized group survived the lethal challenge with the JHA1 strain (Figure 4F). Taken together, our results demonstrate that i.c. inoculation of the JHA1 strain allowed the determination of protective immunity induced in immunocompetent mice submitted to vaccination with anti-DENV vaccines capable of mounting cellular-based immune responses to NS proteins.

## Discussion

Dengue fever is currently the main human arboviral infection in several countries, for which there are no safe, effective and widely available vaccines or antiviral drugs in clinical use. Dengvaxia®, the only DENV vaccine approved for use in humans, has multiple drawbacks, including lack of efficacy in DENV-naïve individuals and higher incidence of severe dengue disease cases in some vaccinated subjects relative to in unvaccinated individuals, which supports a role for Abs in DENV pathogenesis (ADE) (25–31). On the other hand, DENV NS proteins were shown to be good targets for T cell-based immune responses after DENV infection (38–41). The present study reported the development and evaluation of a vaccine formulation composed of the main DENV NS proteins encoded by DNA plasmids, which was tested with a DENV experimental infection model in which a nonadapted neurovirulent strain of DENV2 successfully infected and killed B6 WT mice through the induction of encephalitis. Immunization with each vaccine plasmid correlates with IFN-γ production by spleen and brain-infiltrating monocytes cells, with no detection of antigen-specific IgG antibodies. Furthermore, immunization with a combination of these three antigens encoded by the DNA vaccine mix induced a T cell response that protects against a lethal challenge with the DENV2 JHA1 strain in B6 WT mice. These results suggest that prevention of mouse death was associated with vaccine-induced brain-infiltrating IFN-γ-producing T cells. Altogether, the present study indicates that targeting DENV NS proteins as main vaccine targets is a relevant aspect to be considered in the development of safe and effective anti-dengue vaccines.

The lack of an ideal murine model for DENV infection is often mentioned as a main obstacle to access relevant features of DENV pathogenesis, which delays the development of effective vaccines and antiviral drugs (12, 13, 15). The innate immune system inhibits DENV replication in mice, and as a result, the vast majority of mouse models presently used for the study of DENV infections rely mainly on immunodeficient mice that lack key components of the immune responses or on murine-adapted virus strains (11, 13). JHA1 is a nonadapted DENV2 strain described as naturally neurovirulent to BALB/c mice and displays several single nucleotide polymorphisms with regard to other DENV2 isolates that may explain the neurovirulence in immunocompetent mice even at low MOI numbers (18). Nonetheless, the intracranial inoculation route, although extensively used for testing mouse adapted DENV strain in immunocompetent mouse model for dengue (16, 19, 20, 50, 53), does not represent a natural infection route and is, therefore, a major drawback. However, neurological manifestations have been reported by several research groups, including encephalitis, meningitis, and myelitis in patients with or without warning signs, regardless of the viral serotype (58–62). Thus, DENV i.c. inoculation in mice provides a straightforward readout parameter for vaccine evaluation and may be a useful approach to gain insights into the viral and immune mechanisms involved in atypical dengue-induced neurological complications in patients (63).

Consistent with our previous reports, JHA1 is capable of successfully replicating in the mouse central nervous system (CNS). In fact, we observed an increase in viable viral particles in infected brains, suggesting that JHA1 successfully replicates in this organ. The increase in viral loads is followed by body weight loss, CNS impairment signs (i.e., paralysis), and increased serum markers of tissue damage, implying that systemic and brain-specific aspects of viral infection are involved in the DENV-induced pathogenesis observed. Furthermore, the histopathological features of inflammation and the increase in brain-infiltrating lymphocytes support the idea of an ongoing peripheral cellular immune response (63–65). Furthermore, we have demonstrated that depletion of either CD8^+^ or CD4^+^ T cells prior to challenge impairs protection in DENV2-primed BALB/c mice. Indeed, based on the JHA1 model, we have supported previous clinical evidence that DENV2-specific IFN-γ-producing CD8^+^ T cells target mainly the NS 1, 3, and 5 proteins (37, 66).

It is well known that both CD8^+^ and CD4^+^ T cells play a major role in protection against DENV and other flavivirus infections in different experimental models (32, 37, 67–69). Since mouse and human studies have demonstrated that T cells broadly target HLA-restricted epitopes of NS proteins (47, 70), we constructed DNA vaccines coding the full NS 1, 3, and 5 proteins. As expected, immunization with each plasmid, or their combination, induced immune responses against these antigens, as measured by the number of spleen and brain-infiltrating monocytes cells producing IFN- γ after *ex vivo* stimulation with peptides derived from the respective NS proteins. On the other hand, immunized mice did not produced detectable levels of antigen-specific IgG, probably due the lack of a signal peptide in the DNA construction, resulting in production of the target recombinant protein only intracellular which may hamper the gathering of B cells with the vaccine targets, resulting in no antibody production (71). These results are in agreement with several previous observations showing that DNA vaccines encoding NS proteins induce Th1-biased immune responses that correlate with different degrees of protection in different mouse models (19, 20, 52, 72). Since type I interferon is known to protect mice from fatal neurotropic infection with several flaviviruses through systemic and local antiviral responses (73–78), and IFN-γ-producing CD8^+^ T cells are required for DENV clearance from the CNS (79), our results offer evidences that this mechanisms is directly involved in the observed protection. Furthermore, DENV-specific CD8^+^ T cells prevent ADE (32), and are essential for cross-protection against secondary infection with heterotypic DENV and also ZIKV (35, 80). Hence, an anti-DENV vaccine based on NS proteins that can activate such cells, are safer and more prone to induce cross-reactive protective immunity against primary dengue infection and to prevent pathogenic effects of sub-neutralizing antibodies during heterologous DENV serotype infection. However, since in our model virus seems to replicate exclusive in the brain and the this organ has a low uptake from circulating IgG (81), our model may not be suitable to test whether the ADE phenome can be prevent by vaccine-induced CD8^+^ T cells. Further experiments are required to answer such relevant point regarding the development of dengue vaccines.

Upon i.c. viral challenge and the consequent virus-induced tissue damage, brain inflammatory responses result in upregulation of adhesion molecules in endothelial cells, which attract antigen-specific T cells to infiltrate the brain (82). In the present study, we demonstrated that immunization with pNS (1,3,5) resulted in an average of a 5-fold increase in the number of brain-infiltrating inflammatory cells. During infection, viral-derived peptides are expressed in the context of MHC class I molecules by infected neural cells or in the context of both MHC classes by antigen-presenting cells (microglia). In this inflammatory environment, T cells are activated to deploy antiviral activities (83). In this sense, more experiments are necessary to determine whether vaccine-induced T cells are capable to proliferate and migrate upon secondary viral antigen exposure. IFN-γ-mediated immune responses are described as crucial for viral clearance in DENV-infected mice, both for the early and late stages of infection, while TNF and IL-6 are associated with severe dengue in mice and humans (55, 84, 85). We demonstrated that vaccinated mice successfully mounted a protective immune response against DENV infection, that is probably based on IFN-γ production by both splenic and brain-infiltrating mononuclear cells. On the other hand, mock-immunized mice responded to viral challenge producing TNF, IL-6, and IL-10. The pattern of cytokines produced upon DENV challenge correlated with the survival rates, i.e., vaccine-induced IFN-γ correlated with protection, while the lack of sufficient IFN-γ production and increased levels of disease-associated cytokines correlated with a failure to induce protection in this experimental DENV model.

In summary, our results demonstrate that vaccination with DNA vaccines encoding NS proteins elicits robust cellular responses that promote protection against DENV2 infection in an IFNγ-dependent manner in immunocompetent mice. These findings validate and extend the use of the JHA1-based model for infection in B6 WT mice and provide significant insights into the development or evaluation of a preventive vaccine strategy against DENV infection. Furthermore, our study further supports the relevance of comprehensive analyses of T cell responses to nonstructural proteins as a key aspect involved in DENV vaccine-elicited protection. This point shall be considered for future clinical trials and may open the way to the development of safe and efficient vaccines against DENV and other flaviviruses.

## Data Availability Statement

The original contributions presented in the study are included in the article/supplementary material, further inquiries can be directed to the corresponding author/s.

## Ethics Statement

The animal study was reviewed and approved by Institutional Animal Care and Use Committee (CEUA) of the University of São Paulo (protocol number 14/2016).

## Author Contributions

RA, CF, JA, and LF: conceptualization. RA, RA-S, DF-M and SP: data curation. RA, LP, and MR-J formal analysis. LF and JP: funding acquisition. RA, RA-S, LP, MR-J, SP, and AC: investigation. RA, LF, and CF: methodology. RA and LF: project administration. NS, LF, and JP: resources. RA and LF: supervision. RA and NS: validation. RA and CF: visualization. RA and LF: writing—original draft. RA, JP, JA, and LF: writing—review and editing. All authors contributed to the article and approved the submitted version.

## Conflict of Interest

The authors declare that the research was conducted in the absence of any commercial or financial relationships that could be construed as a potential conflict of interest.
