# Corrigendum: Protective Immunity to Dengue Virus Induced by DNA Vaccines Encoding Nonstructural Proteins in a Lethal Challenge Immunocompetent Mouse Model

**DOI:** 10.3389/fmedt.2020.626114

**Published:** 2020-11-27

**Authors:** Rúbens Prince dos Santos Alves, Robert Andreata-Santos, Carla Longo de Freitas, Lennon Ramos Pereira, Denicar Lina Nascimento Fabris-Maeda, Mônica Josiane Rodrigues-Jesus, Samuel Santos Pereira, Alexia Adrianne Venceslau Brito Carvalho, Natiely Silva Sales, Jean Pierre Schatzmann Peron, Jaime Henrique Amorim, Luís Carlos de Souza Ferreira

**Affiliations:** ^1^Laboratório de Desenvolvimento de Vacinas, Departamento de Microbiologia, Instituto de Ciências Biomédicas, Universidade de São Paulo, São Paulo, Brazil; ^2^Laboratório de Interações Neuroimunes, Departamento de Imunologia, Universidade de São Paulo, São Paulo, Brazil; ^3^Laboratório de Microbiologia, Centro das Ciências Biológicas e da Saúde, Universidade Federal Do Oeste da Bahia, Barreiras, Brazil

**Keywords:** mouse model, dengue, nonstructural proteins, DNA vaccines, IFN-γ

An author name was incorrectly spelled as “**Mônoca Josiane Rodrigues-Jesus**”. The correct spelling is “**Mônica Josiane Rodrigues-Jesus**”.

The authors apologize for this error and state that this does not change the scientific conclusions of the article in any way. The original article has been updated.

